# DISCOVER: Dutch Iliac Stent trial: COVERed balloon-expandable versus uncovered balloon-expandable stents in the common iliac artery: study protocol for a randomized controlled trial

**DOI:** 10.1186/1745-6215-13-215

**Published:** 2012-11-19

**Authors:** Joost A Bekken, Jan Albert Vos, Ruud A Aarts, Jean-Paul PM de Vries, Bram Fioole

**Affiliations:** 1 Department of Vascular Surgery, Maasstad Ziekenhuis, Maasstadweg 21, Rotterdam, 3079 DZ, The Netherlands; 2 Department of Interventional Radiology, St. Antonius Ziekenuis, Koekoekslaan 1, Nieuwegein, 3435 CM, The Netherlands; 3 Department of Interventional Radiology, Maasstad Ziekenhuis, Maasstadweg 21, Rotterdam, 3079 DZ, The Netherlands; 4 Department of Vascular Surgery, St. Antonius Ziekenuis, Koekoekslaan 1, Nieuwegein, 3435 CM, The Netherlands

**Keywords:** Peripheral arterial occlusive disease, Atherosclerotic disease, Common Iliac Artery, Intermittent Claudication, Critical Limb Ischemia, Endovascular, Stenting, Covered stent

## Abstract

**Background:**

Iliac artery atherosclerotic disease may cause intermittent claudication and critical limb ischemia. It can lead to serious complications such as infection, amputation and even death. Revascularization relieves symptoms and prevents these complications. Historically, open surgical repair, in the form of endarterectomy or bypass, was used. Over the last decade, endovascular repair has become the first choice of treatment for iliac arterial occlusive disease. No definitive consensus has emerged about the best endovascular strategy and which type of stent, if any, to use. However, in more advanced disease, that is, long or multiple stenoses or occlusions, literature is most supportive of primary stenting with a balloon-expandable stent in the common iliac artery (Jongkind V et al., J Vasc Surg 52:1376-1383,2010). Recently, a PTFE-covered balloon-expandable stent (Advanta V12, Atrium Medical Inc., Hudson, NH, USA) has been introduced for the iliac artery. Covering stents with PTFE has been shown to lead to less neo-intimal hyperplasia and this might lower restenosis rates (Dolmatch B et al. J Vasc Interv Radiol 18:527-534,2007, Marin ML et al. J Vasc Interv Radiol 7:651-656,1996, Virmani R et al. J Vasc Interv Radiol 10:445-456,1999). However, only one RCT, of mediocre quality has been published on this stent in the common iliac artery (Mwipatayi BP et al. J Vasc Surg 54:1561-1570,2011, Bekken JA et al. J Vasc Surg 55:1545-1546,2012). Our hypothesis is that covered balloon-expandable stents lead to better results when compared to uncovered balloon-expandable stents.

**Methods/Design:**

This is a prospective, randomized, controlled, double-blind, multi-center trial. The study population consists of human volunteers aged over 18 years, with symptomatic advanced atherosclerotic disease of the common iliac artery, defined as stenoses longer than 3 cm and occlusions. A total of 174 patients will be included.

The control group will undergo endovascular dilatation or revascularization of the common iliac artery, followed by placement of one or more uncovered balloon-expandable stents. The study group will undergo the same treatment, however one or more PTFE-covered balloon-expandable stents will be placed. When necessary, the aorta, external iliac artery, common femoral artery, superficial femoral artery and deep femoral artery will be treated, using the standard treatment.

The primary endpoint is absence of binary restenosis rate. Secondary endpoints are reocclusion rate, target-lesion revascularization rate, clinical success, procedural success, hemodynamic success, major amputation rate, complication rate and mortality rate. Main study parameters are age, gender, relevant co-morbidity, and several patient, disease and procedure-related parameters.

**Trial registration:**

Dutch Trial Register, NTR3381.

## Background

Peripheral Arterial Occlusive Disease (PAOD) is a disease defined as reduced arterial blood flow to the lower extremities due to atherosclerotic arterial lesions and is diagnosed by an ankle-brachial index less than 0.9. It may lead to intermittent claudication (IC) or, with progression of the disease, critical limb ischemia (CLI). Only one out of every four to five patients with PAOD will be symptomatic [1]. The most common clinical manifestation of PAOD is intermittent claudication involving the pelvis, upper thigh and lower limb. It is defined as ischemic pain occurring during exercise, which is quickly relieved with rest (Fontaine II, Rutherford 1 to 3). CLI is a more severe presentation of PAOD, defined as ischemic rest pain (Fontaine III, Rutherford 4) or ischemic skin lesions: either ulcers or gangrene (Fontaine IV, Rutherford 5 and 6, respectively) [1]. See Table 1 for a description of the Rutherford classification. Patients presenting with CLI usually have multisegmental disease with involvement of the infra-inguinal arteries [2]. Ten to twenty percent of patients with IC will progress to CLI in the course of their disease [3-6]. The most important risk factors for progression to the advanced form of PAOD are age, tobacco use and diabetes mellitus [6].

**Table 1:** Overview of the Rutherford-classification for PAOD

**Grade**	**Category**	**Clinical description**	**Objective criteria**
0	0	Asymptomatic - no hemodynamically significant occlusive disease	Normal treadmill test and ABI ≥0.9
	1	Mild claudication	Completes treadmill test. AP after exercise >50 mmHg, but at least 20 mmHg lower than resting value
I	2	Moderate claudication	Between categories 1 and 3
	3	Severe claudication	Cannot complete treadmill test *and* AP after exercise <50 mmHg
II	4	Ischemic rest pain	Resting AP <40 mmHg, flat or barely pulsatile ankle or metatarsal PVR, TP <30 mmHg
III	5	Minor tissue loss - nonhealing ulcer, focal gangrene with diffuse pedal ischemia	Resting AP <60 mmHg, ankle or metatarsal PVR flat or barely pulsatile, TP <40 mmHg
	6	Major tissue loss - extending above TM level, functional foot no longer salvageable	Same as category 5

AP = Ankle pressure, PAOD = Peripheral Arterial Occlusive Disease, PVR = pulse volume recording, TP = Toe pressure.

### Epidemiology

Newman *et al*. [7] described the prevalence of PAOD (asymptomatic and symptomatic) in the general population. They found a prevalence of 13.4% in those over 65 years of age, rising to 21.6% in those over 75 years of age. The German getABI study [8] showed a prevalence of 19.8% in men over 65, and 16.8% in women over 65. The exact overall incidence of PAOD is not known, but the Framingham Study showed an incidence of IC of 26/10,000 in men and 12/10,000 in women [9]. Anatomically, approximately 30% of the arterial lesions in PAOD are located in the iliac arteries [6].

### Endovascular treatment

In 1964, one year before the technique was used for coronary arteries, the endovascular approach for treating aorto-iliac lesions was introduced by Charles Dotter [10]. In 1974, a catheter-mounted inflatable balloon that could fit over a guidewire was developed by Andreas Grüntzig, significantly improving the technique [11]. Finally, in 1985, the first intraluminal stent was developed by Julio Palmaz, further improving the results of endovascular treatment [12].

Despite the introduction of endovascular treatment in 1964, open surgical treatment has long been the treatment of choice. Open surgical repair provides good long-term patency (IC: 85 to 92%, CLI: 78 to 83%) [13]. The perioperative morbidity and mortality, however, is substantial [13-17]. Due to ongoing improvements in materials and techniques over the past decades, endovascular techniques for aorto-iliac obstuctions have more and more replaced open surgical repair. These minimally invasive techniques show reduced morbidity and mortality when compared to open surgery [18-21].

### TASC guidelines

**Figure 1:**
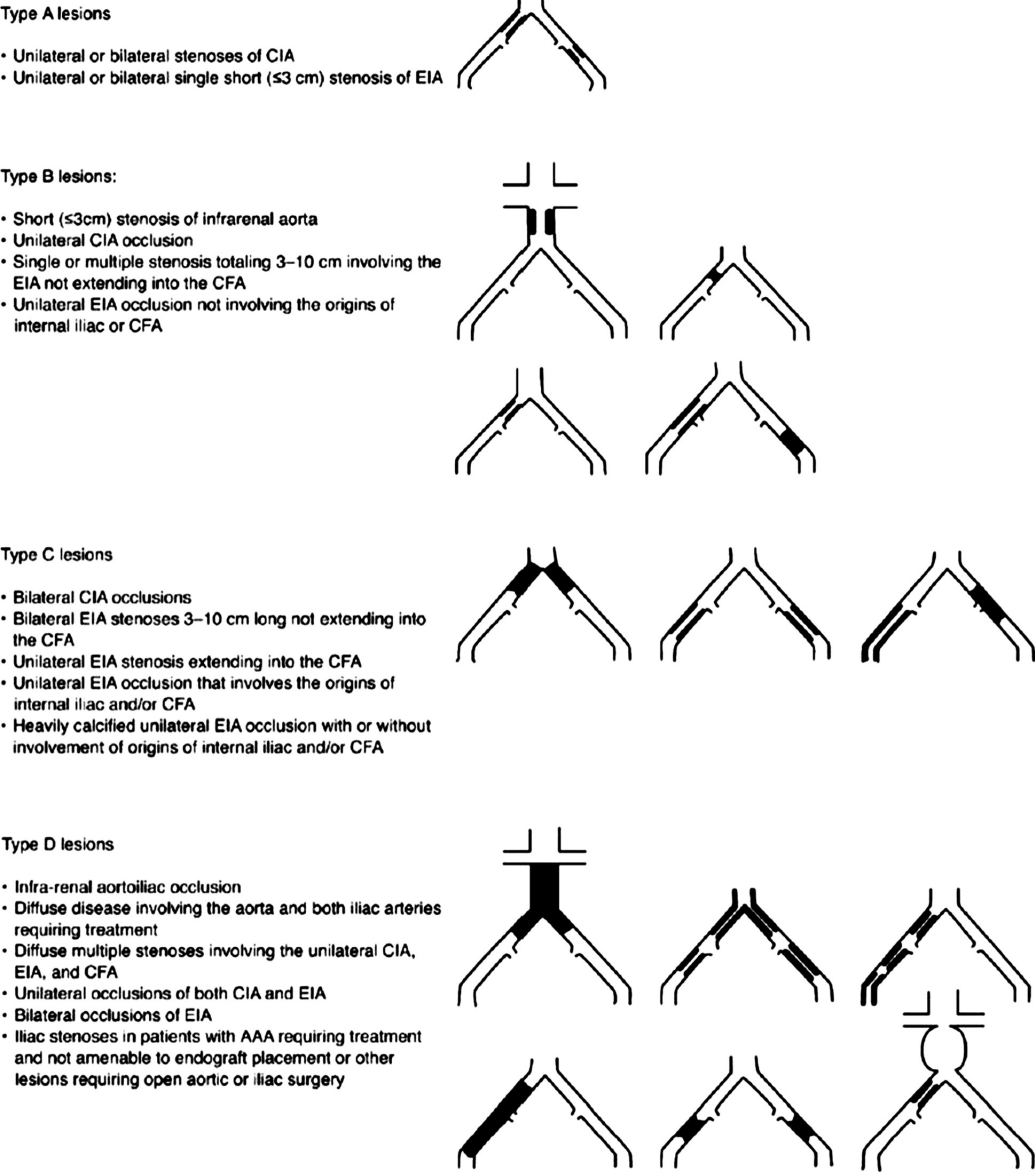
The TASC II classification of aortoiliac lesions.

Aorto-iliac Occlusive Disease (AIOD) is classified by the Trans Atlantic Inter-Society Consensus on the Management of Peripheral Arterial Disease (TASC II). This classification divides AIOD into four types (see Figure 1), based on the amenability to endovascular repair. The current TASC guidelines were published in 2007 [1] and recommend an endovascular approach in type A and B lesions, and an open surgical approach for type C and D lesions. Endovascular repair is only recommended in patients with type C lesions, who have a low healing potential following surgical revascularization. However, a recent meta-analysis was published by Jongkind *et al*. reporting on endovascular treatment for extensive AIOD (TASC C and D lesions). A total of 19 non-randomized cohort studies, containing 1,711 patients were included. Although 4- and 5-year primary patency rates for endovascular repair were lower compared to open surgical treatment (60 to 86%), the secondary patency rates were comparable (80 to 98%), with most reinterventions performed endovascularly. Two studies retrospectively compared open and endovascular therapy. Significantly lower perioperative morbidity and shorter hospital stay was reported in the endovascular group. Three- and four-year primary patency rates were also significantly lower for the endovascular group (69% vs 93%, *P* = 0.013 and 74% vs 93%, *P* = 0.002), however secondary patency was comparable with surgical repair (89% vs 100%, *P* >0.05 and 95% vs 97%, *P* = 0.3) [22,23].

### PTA or PTA with stenting for iliac obstructions

When treating AIOD endovascularly, there has been some debate on whether to stent all lesions (‘direct’ or ‘primary’ stenting) or to perform balloon dilatation and only place a stent on indication, such as dissection or residual stenosis (‘selective’ stenting). A meta-analysis performed by Bosch *et al*. [24] in 1997 compared these two methods. A total of 1,300 selective stent patients (6 studies) and 816 direct stent patients (8 studies), were compared. This study showed higher technical success and patency rates after direct stenting. However, a randomized controlled trial conducted by the same group, published in 1998, showed, after 5 to 8 years of follow-up, no significant difference in iliac patency and ABI. A small, but significant, difference in symptomatic success was found, in favor of selective stenting [25,26]. These results are equivocal, due to a low technical success rate (approximately 80%) and the exclusion of advanced disease. Since then, an overwhelming amount of studies have shown favorable results of direct stenting, especially in TASC C and D lesions [2,20,22,23,27-40]. In the aforementioned review by Jongkind *et al*., 15 out of 19 included studies employed direct stenting rather than selective stenting. Therefore, evidence for direct stenting in simple disease is lacking compared to more advanced disease, where literature supports the employment of this strategy.

### Distal outflow after endovascular repair

Several studies have shown that decreased outflow leads to lower patency rates and that, when treating the iliac artery endovascularly, sufficient outflow from the common femoral artery is mandatory [37,41].

### Balloon-expandable or self-expanding stents for iliac obstructions

Balloon-expandable stents are usually made of stainless steel, while self-expanding stents are usually made of Nitinol, an alloy of nickel and titanium. Nitinol is a ‘thermal memory’ alloy which can be heat-treated to assume a predetermined shape above a set temperature, which in this case is 30°C. Balloon-expandable stents are characterized by much greater radial strength, compared to self-expanding stents. Self-expanding stents, on the other hand, have greater elasticity, allowing them to regain their shape after the compressing force reduces. Another advantage of self-expanding stents is their higher trackability, meaning they are easier to maneuver through tortuous vessels or past the aortic bifurcation in the contralateral approach. Balloon-expandable stents generally have higher radiopacity, which facilitates accurate placement [42]. Due to its greater flexibility and elasticity, self-expanding stents are advised in tortuous vessels, over joints and in mechanically exposed vessels (subclavian and popliteal arteries). On the other hand, in rigid and straight lesions balloon-expandable stents are generally considered more appropriate [43,44].

### Bare or covered stents for iliac obstructions

One of the main causes for late failure after stent placement is in-stent stenosis, predominantly caused by neo-intimal hyperplasia [45]. The wall injury inherent to any vascular intervention leads to the exposure of underlying atheromatous tissue and the elution of macromolecules, among them pro-inflammatory mediators. These attract macrophages that transmigrate into the vascular wall to release further cytokines, metalloproteinase, and growth factors that play a role in initiating the restenotic process. Therefore, endoluminal sealing of the vessel with a membrane covered stent may potentially inhibit restenosis formation [46]. The stent-covering materials are the same that have been used for bypass grafts: polyethylene terephthalate (PET, brand name Dacron) and polytetrafluoroethylene (PTFE, brand name Teflon). Of these two frequently used fabrics, PET has the downside of being more thrombogenic than PTFE [47]. In concordance with this, studies investigating Dacron-covered stent grafts in iliac and femoral arteries have shown disappointing results [48-50]. Therefore, current research has been focused on PTFE-covered stents. PTFE for vascular prosthetic applications is known as expanded polytetrafluoroethylene (ePTFE). Expansion is part of the manufacturing process to modify the solid material into a porous lattice. When PTFE is used to cover a balloon-expandable metal stent, the material dilates simultaneously with the stent, with a resulting decrease in the material wall thickness [45].

Several studies have been published describing the results of PTFE-covered stents in PAOD of the iliac artery. These are shown in Table 2.

**Table 2:** Results of studies reporting on the results of PTFE-covered stents for PAOD in the iliac artery

**Author, year**	**Type of study**	**Number**	**Type of stent**	**Freedom of binary restenosis**	
				**Covered**	**Uncovered**
Sabri, 2010 [54]	Retrospective, comparative	26 covered	Balloon-expandable	1 year: 92%	1 year:78%,
		28 uncovered		2 year:92%,	2 year: 62%
Lammer, 2000 [61]	Prospective	61	Self-expanding	6 month: 98%,	
				1 year: 91%	
Wiesinger, 2005 [62]	Prospective	60	Self-expanding	6 month: 94%,	
				1 year: 91%	
Bosiers, 2007 [63]	Prospective	91	Balloon-expandable	1 year: 91%	
Chang, 2008 [53]	Retrospective, comparative	71 covered	Mostly self-expanding	5 year: 87%	5 year: 53%
		122 uncovered			
Mwipatayi, 2011	Randomized controlled trial	83 covered	Both	18 month: 92%	18 month: 75%
		84 uncovered			

As shown in Table 2, primary patency rates of covered stents are excellent. However, most studies are either retrospective or do not have a control group. Only one randomized controlled trial has been performed, which is the COBEST-trial (Covered versus Balloon Expandable Stent Trial). This trial compares the Advanta V12 balloon-expandable PTFE-covered stent versus uncovered stents. In this trial, various types of uncovered stents are used, including self-expanding. Since balloon-expandable and self-expanding stents have very different characteristics, this is likely to influence the results. Some of the other drawbacks of this study include usage of the TASC-classification for sub-group analysis, and making no distinction between the CIA and the EIA. The TASC-classification is a very general and heterogenous classification, making it useful for day to day practice, but not so much for research purposes. Categorizing subgroups based on specific lesion characteristics (such as occlusion vs stenosis, and length of the lesion), allows for much more accurate analysis. Furthermore, the external and common iliac have very different anatomical characteristics. Where the CIA is a straight and immobile vessel, being fixed to the sacral promontory, the EIA has a much more tortuous course, along with stretching during extension of the hip [51]. To our knowledge, no comparative studies have been published on this topic, but biomechanical studies do confirm our opinion [42]. Furthermore, a retrospective study showed a decreased patency rate after stent placement in the EIA compared to a similar treatment in the CIA [52]. In this trial, we will avoid these flaws, and perform a more methodologically and scientifically solid study, by focusing on just the CIA, and using lesion characteristics, rather than the TASC-classification, to classify lesions.

### Conclusion and rationale of this study

A primary endovascular approach should be attempted in all patients with PAOD and iliac lesions. When performing endovascular repair, there are many different strategies and types of stents to be used, as described above. There is no general consensus on which strategy and type of stent is best for which artery. However, for more advanced disease of the common iliac artery, literature is most supportive of direct stenting with a balloon-expandable stent. A recent innovation in this area is the PTFE-covered stent, which might further improve the primary patency of iliac stenting. However, only one RCT, of mediocre quality and several small cohort studies and retrospective comparative studies on this subject are available. Therefore, the purpose of this study is to compare PTFE covered balloon-expandable stents with non-covered balloon-expandable stents in patients with advanced occlusive disease in the common iliac artery, defined as stenoses longer than 3 cm and occlusions (TASC B, C and D lesions).

## Methods/Design

### Objectives

#### Primary objective

• To assess the absence of binary restenosis rate of advanced atherosclerotic lesions of the common iliac artery with a balloon-expandable PTFE-covered stent (Advanta V12, Atrium Medical Inc., Hudson, NH, USA), when compared to selected balloon-expandable uncovered stents, in a 2-year follow-up period.

#### Secondary objectives

• To assess the reocclusion rate and target-lesion revascularization rate after endovascular treatment of advanced atherosclerotic lesions of the common iliac artery with a balloon- expandable PTFE-covered stent (Advanta V12, Atrium Medical Inc., Hudson, NH, USA), when compared to selected balloon-expandable uncovered stents, in a 2-year follow-up period.

• To assess morphological outcome after endovascular treatment of advanced atherosclerotic lesions of the common iliac artery with a balloon-expandable PTFE-covered stent (Advanta V12, Atrium Medical Inc., Hudson, NH, USA), when compared to selected balloon-expandable uncovered stents, in a 2-year follow-up period.

To assess clinical outcome after endovascular treatment of advanced atherosclerotic lesions of the common iliac artery with a balloon-expandable PTFE-covered stent (Advanta V12, Atrium Medical Inc., Hudson, NH, USA), when compared to selected balloon- expandable uncovered stents, in a 2-year follow-up period.

• To assess hemodynamic outcome after endovascular treatment of advanced atherosclerotic lesions of the common iliac artery with a balloon expandable PTFE-covered stent (Advanta V12, Atrium Medical Inc., Hudson, NH, USA), when compared to selected balloon- expandable uncovered stents, in a 2-year follow-up period.

• To assess the complication rate, mortality rate and amputation rate after endovascular treatment of advanced atherosclerotic lesions of the common iliac artery with a balloon-expandable PTFE-covered stent (Advanta V12, Atrium Medical Inc., Hudson, NH, USA), when compared to selected balloon-expandable uncovered stents, in a 2-year follow-up period.

### Study Design

A prospective, randomized, controlled, double-blind, multi-center trial with a follow-up period of 2 years.

Centers currently participating are:

• Maasstad Hospital, Rotterdam, The Netherlands

• Sint Antonius Hospital, Nieuwegein, The Netherlands

More centers may be added during the course of the trial.

### Study population

Patients with symptomatic, advanced atherosclerotic lesions of the common iliac artery presenting at one of the participating hospitals.

#### Inclusion criteria

• Age over 18;

• Symptomatic, atherosclerotic lesion of the common iliac artery, either a hemodynamically significant stenosis with a length of more than 3 cm, or an occlusion. This will be measured on the pre-dilation DSA-images, where a diameter reduction of >50% is considered significant. A hemodynamically significant stenosis is confirmed with an intra-arterial translesional systolic blood pressure gradient measurement, where >10 mmHg pressure gradient is considered significant. Serial lesions less than 2 cm apart will be regarded as one long stenosis;

• Signed informed consent form.

#### Exclusion criteria

• Stenosis with a length of less than 3 cm;

• Presence of a metastatic malignancy, or other disease that limits life expectancy to less than 2 years;

• Previous endovascular or surgical treatment of the common iliac artery on the affected side;

• Inability or unwillingness to comply with the follow-up schedule;

• Mental disability or language barrier that hinders the ability to understand and comply with the informed consent;

• Pregnancy or breast-feeding;

• Severe renal failure (e-GFR <30 mL/min/1.73 m^2^);

• Known allergy to iodinated contrast agents or to PTFE;

• Contra-indication for anti-coagulation;

• Acute limb ischemia;

• Occlusion of the abdominal aorta;

• Aneurysm of the abdominal aorta that is not amenable to endograft placement.

#### Sample size calculation

Based on the available literature [53,54], we expect a 2-year absence of binary restenosis rate of 90% in the PTFE-covered stent group, and a 2-year absence of binary restenosis rate of 72% in the uncovered stent group. Based on the log rank test for equality of survival curves, with an alpha error level of 0.05 and a beta error level of 0.2, 79 patients per group will be necessary. We anticipate a loss to follow-up of less than 10%, so we will include 87 patients per group, for a total of 174 patients. This calculation was performed using nQuery Advisor (Statistical Solutions Ltd., Farmer’s Cross, Ireland). The combined participating centers perform over 200 endovascular procedures for advanced iliac PAOD annually. With an estimated participation rate of 75%, estimated inclusion time is 2 years.

No sample size calculation was performed for the subgroups as there is no data available on freedom of binary restenosis rates for these groups, and therefore no well-founded calculation can be made.

### Treatment of subjects

The aim of the treatment is to obtain a patent aorta, common and external iliac artery and a patent common femoral artery with outflow via at least the superficial or deep femoral artery. Therefore, not only the common iliac artery will be treated, but if indicated, the aorta, external iliac artery and the common femoral artery will be treated additionally. This strategy corresponds with daily practice.

The common iliac artery will be treated with PTA and direct stent placement. Patients will be randomized to either the Advanta V12 PTFE-covered stent (Atrium Medical Inc., Hudson, NH, USA) or a balloon-expandable uncovered stent (see ’Investigational product’). If the aorta needs additional treatment, the physician is free to use the following techniques: PTA or PTA and balloon-expandable stent placement. If the external iliac artery needs additional treatment, the physician is free to use the following techniques: remote endarterectomy, PTA or PTA and self-expandable uncovered stent placement. If the common femoral artery needs additional treatment, an endarterectomy is indicated. Patients will be treated under local anesthesia for totally endovascular procedures and under regional or general anesthesia if an additional endarterectomy is warranted.

#### Treatment of the common iliac artery

A 6F or 7F sheath (depending on the type and diameter of the stent) is introduced into the ipsilateral common femoral artery. If necessary an adjunctive contralateral approach may be used at the discretion of the physician, either to make angiographic images, or to facilitate passage of the diseased segment. All patients receive 5,000 units of unfractionated heparin. A diagnostic catheter is introduced into the aorta over a 0.035-inch guidewire. Digital Subtraction Angiography (DSA) is performed and saved according to protocol. In stenoses, the pressure gradient is measured using a 70cm straight catheter (4F) without side holes, which is withdrawn through the stenosis. A translesional systolic blood pressure gradient of >10mmHg is considered significant. The pressure gradient, as well as absolute pressures, are documented. The length of the diseased segment is measured per-operatively on the DSA-images, and based on the pressure gradient and length measurement the final decision is made whether or not to include and randomize the subject. Any occlusion is included, as well as any stenosis longer than 3 cm. Serial lesions less than 2 cm apart will be regarded as one long stenosis and will be randomized.

Based on randomisation, an Advanta V12 stent or a balloon-expandable uncovered stent is placed. A stenosis should be fully covered by the stent, including 5mm proximal and distal to the stenosis, based on the inflated length of the stent. In case of proximal lesions, in which the stent would extend into the aorta, and in distal lesions, in which the stent would cover the internal iliac artery (IIA), this 5mm margin is not mandatory. In case of a stenosis including the distal aorta or iliac bifurcation, ‘kissing stents’ will be placed, which will extend into the aorta. In case of a stenosis extending into the EIA, the lesion in the CIA will be treated with a balloon-expandable stent. The stenotic segment extending into the EIA will be treated with PTA alone, PTA and a self-expanding stent or remote endarterectomy. We try to avoid covering the IIA with a stent.

In case of recanalization of a total occlusion, at least the recanalized segment and any adjoining stenoses should be fully covered by the stent, including 5mm proximal and distal to the stenosis. The diameter will be measured on the DSA-images, based on the diameter of a healthy segment on the ipsilateral side. In case there is no healthy segment, or in case of occlusion, the contralateral CIA will be used for measurement. In case of bilateral occlusion, the pre-operative CT-angiography or MR-angiography will be used to measure the CIA diameter. After measurement of the common iliac artery diameter all stents are a maximum of 0.5mm oversized. The balloon is longer than the stent and extends out of the stent. When inflating the balloon, the shape of the balloon has some resemblance to a dog bone. If the balloon is oversized more than 0.5mm, the expanding balloon outside the stent is thought to damage the artery wall, causing local activation and progression of the atherosclerotic process at the edge of the stent.

After stent placement a completion DSA is performed according to protocol and saved, and the pressure gradient is once again measured and documented, as described previously. Less than 30% residual diameter reduction of the treated lesion and a systolic pressure gradient of less than 5 mmHg will be considered a technical success. The stent location will be documented by describing the exact distance from the aortic bifurcation and the iliac bifurcation. This will ease localization of the stent on DUS during follow-up.

After removal of the sheath(s) a closure device may be used.

#### Treatment of the aorta

An additional hemodynamically significant stenosis in the aorta must be treated by either PTA or PTA and balloon-expandable uncovered stent placement. For the endovascular treatment of the aorta, as far as we know, there is no evidence, which supports direct stenting. Therefore, we consider PTA, with stenting only on indication, the standard treatment. The PTA balloon and stent should be 1mm oversized.

#### Treatment of the external iliac artery

An additional hemodynamically significant stenosis or occlusion in the external iliac artery must be treated by either remote endarterectomy, PTA or PTA and self-expandable uncovered stent placement. We believe that for the EIA self-expandable stents are more appropriate, due to the tortuosity of the artery and its movement during flexion of the hip. As for the aorta, for the EIA there is also no evidence as far as we know, which supports direct stenting. Of course, since these two types of stents have a very different biomechanical behaviour, evidence on the use of balloon-expandable stents should not be extrapolated to self-expanding stents. The PTA balloon and stent should be 1mm oversized. After recanalization of an occluded external iliac artery PTA and additional self-expandable uncovered stent placement is warranted.

#### Treatment of the common femoral artery

A hemodynamically significant stenosis or occlusion of the common femoral artery must be treated by endarterectomy. This procedure will be combined with treatment of the common iliac artery and, if indicated, external iliac artery. If there is no appropriate outflow from either the superficial or deep femoral artery, the physician may choose to obtain sufficient outflow by using remote endarterectomy, recanalisation with or without stent placement, or bypass.

#### Medication

All patients receive Aspirin 100mg daily and Simvastatin 40mg daily, indefinitely, starting at least one day prior to the procedure. During the intervention all patients receive 5,000 units of Heparin. After the intervention all patients receive Clopidogrel 75mg daily for a period of 4 weeks.

### Investigational product

#### Name and description of investigational product

The Advanta V12 stent (Atrium Medical Inc., Hudson, NH, USA) is a balloon-expandable stainless steel stent that is fully encapsulated in two layers of PTFE. The PTFE has a porosity of 100 to 120 μm. The Advanta V12 covered stent is available in diameters of 5 to 10 mm. Available stent lengths are 16, 22, 38, and 59 mm. All V12 stents are 0.035-inch guidewire compatible and are pre-mounted on a 7F non-compliant balloon catheter with gold markers embedded at the ends of the balloon. Available catheter lengths are 80 and 120 cm. It is the only balloon-expandable PTFE-covered stent that is registered for use in the iliac artery in Europe.

#### Name and description of control group products

In the control group, several uncovered balloon-expandable stents have been selected to choose from at the physician’s discretion. These stents are currently used in the participating centers.

Omnilink Elite stent (Abbott Laboratories, North Chicago, IL, USA). The Omnilink Elite is a balloon-expandable CoCr (cobalt chromium alloy) stent. It is available in diameters of 4 to 10 mm. Available lengths are 12, 16, 19, 29, 39 and 59 mm. All Omnilink Elite stents are 0.035-inch guidewire compatible and are pre-mounted on a 6F dual layer compliant balloon catheter. Available catheter lengths are 80 and 135 cm. It is registered for use in the iliac artery in Europe.

Dynamic stent (Biotronik, Berlin, Germany). The Dynamic stent is a balloon-expandable stainless steel stent, with the stent struts coated with an amorphous silicon carbide coating. It is available in diameters of 5 to 10 mm. Available lengths are 15, 25, 38 and 56 mm. All Dynamic stents are 0.035-inch guidewire compatible and are pre-mounted on a 6 or 7F compliant balloon catheter. Available catheter lengths are 80 and 130 cm. It is registered for use in the iliac artery in Europe.

Express LD Iliac stent (Boston Scientific, Natick, MA, USA). The Express LD Iliac is a balloon-expandable stainless steel stent. It is available in diameters of 6 to 10 mm. Available lengths are 20, 30, 40 and 60 mm. All Express LD Iliac stents are 0.035-inch guidewire compatible and are pre-mounted on a 6 or 7F compliant balloon catheter. Available catheter lengths are 75 and 135 cm. It is registered for use in the iliac artery in Europe.

Palmaz Genesis stent (Cordis Corporation, Bridgewater, NJ, USA). The Palmaz Genesis is a balloon-expandable stainless steel stent. It is available in diameters of 4 to 10 mm. Available lengths are 12, 15, 18, 19, 24, 25, 29, 39 and 59 and 79 mm. In the participating centers, we only use 24, 39, 59 and 79 in the common iliac artery. All Palmaz Genesis Iliac stents are 0.035-inch guidewire compatible and are pre-mounted on a 6 or 7F compliant balloon catheter. Available catheter lengths are 80 and 135 cm. It is registered for use in the iliac artery in Europe.

Scuba stent (INVATEC S.p.A, Roncadelle, Italy). The Scuba stent is a balloon-expandable CoCr (cobalt chromium alloy) stent. It is available in diameters of 5 to 10 mm. Available lengths are 18, 30, 37, 55 and 75 mm. All Scuba stents are 0.035-inch guidewire compatible and are pre-mounted on a 5F compliant balloon catheter. Available catheter lengths are 80 and 130 cm. It is registered for use in the iliac artery in Europe.

### Endpoints/parameters

Definitions of endpoints and parameters are in concordance with the proposed definitions by the DEFINE group, a multidisciplinary team from various specialties involved in PAOD therapies, from Europe and the USA, which has made a case for more standardized reporting in studies for endovascular treatment of PAOD [55,56].

#### Primary endpoint

• Absence of binary restenosis rate. Defined as the percentage of limbs with absence of hemodynamically significant obstruction in the target-lesion after endovascular treatment.

#### Secondary endpoints

• Reocclusion rate. Defined as complete occlusion of the initially treated target-lesion.

• Target-lesion revascularization (TLR) rate. Defined as the rate and frequency of the need for repeated procedures (endovascular or open surgical) due to a problem arising from the target-lesion (+1 cm proximally and distally to include edge phenomena) in surviving patients with preserved limb.

• Immediate outcome:

– Procedural success. Defined as the combination of technical success, device success and absence of procedural complications.

– Technical success. Defined as successful vascular access and completion of the endovascular procedure and immediate morphological success with less than 30% residual diameter reduction of the treated lesion on completion and a systolic pressure gradient of less than 5 mmHg.

– Device success. Defined as exact deployment of the device, according to the instructions for use, using the assigned device only.

• Clinical outcome:

– Distribution of Rutherford stages during follow-up as compared to baseline.

– Functional outcome:

▪ In patients with IC: Improvement in claudication onset time (COT) and absolute claudicating time (ACT) on standardized treadmill test.

– Improvement in disease-related health status, functioning and quality of life. As defined by the Walking Impairment Questionnaire (WIQ) and RAND-36 questionnaire.

• Hemodynamic outcome:

– Mean and median ABI during follow-up as compared to baseline.

• Morphological outcome:

– Acute diameter gain (change in minimal luminal diameter from base-line to post-intervention.

• Target-extremity revascularization (TER) rate. Defined as the rate and frequency of the need for repeated procedures (endovascular or open surgical) due to a problem arising remote from the target-lesion in surviving patients with preserved limb.

• Mortality rate. Mortality rate associated with the endovascular procedure (that is, mortality within 30 days post-procedure or mortality during a hospitalization >30 days due to the procedure) will be reported separately, as well as overall mortality.

• Complication rate. In contrast with AE’s and SAE’s, which do not need to be related to the treatment, only complications that are (likely) related to the treatment will be reported. This is at the discretion of the investigator.

– Complications will be scored as ‘major’ or minor’. A major complication is a complication that:

▪ Leads to death;

▪ Results in a life-threatening illness or injury;

▪ Results in permanent impairment of a body structure or bodily function;

▪ Requires inpatient hospitalization or prolongation of existing hospitalization;

▪ Results in medical or surgical intervention to prevent permanent impairment to body structure or bodily function.

– Complications will be classified in to four complication categories:

▪ Access site complication (access site including distal to the site): Hematoma/bleeding, arterial/venous occlusion/thrombosis, severe vasospasm, intimal injury/dissection, pseudoaneurysm, arteriovenous fistula, vascular perforation or rupture, arterial embolization distal to puncture site.

▪ Treatment site complication (treatment site including distal to the site): Hematoma/bleeding, arterial/venous occlusion/thrombosis, severe vasospasm, intimal injury/dissection, pseudoaneurysm, arteriovenous fistula, vascular perforation or rupture, arterial embolization distal to treatment site.

▪ Organ-specific complication:

∘ Neurological: TIA, minor and major stroke, seizure;

∘ Cardiovascular: Hypotension or hypertension requiring treatment, arrhythmia requiring treatment, myocardial ischemia/infarction, chronic heart failure;

∘ Respiratory: Profound hypoxia, pulmonary edema, respiratory arrest, pulmonary embolism, pneumothorax;

∘ Gastrointestinal: Gastric bleeding, pancreatitis, peritonitis, abscess, perforation of hollow viscus.

▪ Systemic complication: Allergic/anaphylactic reaction, renal failure, idiosyncratic reaction to drug, fluid/electrolyte imbalance.

• Amputation rate. Divided in to minor (below the ankle) and major (above the ankle). Major amputation is sub-divided in to below-the-knee and above-the-knee. Planned and unplanned amputations will be reported separately. Planned amputations are defined as amputations that were planned prior to the revascularization procedure, that is, when the revascularization procedure is performed to improve the vascularization (and thereby healing potential) of the planned amputation wound.

• Rate of device-specific problems, for example, stent fracture, stent migration.

#### Baseline characteristics

• Risk factors and comorbidities:

– Age;

– Gender;

– Hypertension: Systolic blood pressure ≥140 mmHg and/or diastolic blood pressure ≥ 90 mmHg, or if the patient is on antihypertensieve therapy for the indication of hypertension;

– Hyperlipidemia: LDL-cholesterol >4.4 mmol/L (>170 mg/dL) or triglycerides >2.0 mmol/L (>177 mg/dL), or if the patient is taking lipid-lowering medication for the indication of hyperlipidemia;

– Diabetes mellitus: HbA1c >7% or if the patient consumes oral hypoglycemic agents or uses insulin;

– Smoking: Current smoking status (active/previous/never), number of pack years, number of years since last smoked;

– Ischemic heart disease: History of myocardial infarction, angina pectoris, previous percutaneous or surgical coronary revascularization, positive exercise test, anti-anginal therapy;

– Congestive heart failure: Ejection fraction <40%;

Renal insufficiency: e-GFR <60 mL/min/1.73 m^2;^

– Cerebrovascular disease: Known carotid artery disease and history of minor or major stroke or transient ischemic attack (TIA).

• Medication, pre-, peri- and post-procedural, (including dose, frequency and duration of use):

– Anticoagulants (Acenocoumarol, Fenprocoumon, unfractioned or low molecular weight heparins, and so on);

– Antithrombotic agents (Acetyl salicylic acid, Clopidrogel, Dipyridamol, and so on);

– Statins or other lipid-lowering agents;

– Beta-blockers;

– Angiotensin converting enzyme inhibitors;

– Angiotensin-II receptor antagonists;

– Insulin and oral hypoglycaemic agents;

– Medications for the treatment of IC (Pentoxifylline, Buflomedil).

• Baseline anatomic characteristics of the target lesion in the common iliac artery, as determined by DSA and pre-operative CT-angiography or MR-angiography:

– Arterial inflow: Impaired inflow is defined as presence of hemodynamically significant obstruction in the aorta;

– Arterial outflow: Impaired outflow is defined as presence of hemodynamically significant obstruction in the external iliac artery, common femoral artery or both superficial and deep femoral arteries. Isolated hemodynamically significant obstruction in either the superficial or deep femoral artery will not be considered impaired outflow;

– Reference vessel diameter, obtained from averaging 5mm segments proximal and distal to the lesion;

– Involvement of origin/ostium;

– Lesion length, documented in centimeters and classified as follows:

– Cerebrovascular disease: Known carotid artery disease and history of minor or major stroke or transient ischemic attack (TIA).

• Medication, pre-, peri- and post-procedural, (including dose, frequency and duration of use):

– Anticoagulants (Acenocoumarol, Fenprocoumon, unfractioned or low molecular weight heparins, and so on);

– Antithrombotic agents (Acetyl salicylic acid, Clopidrogel, Dipyridamol, and so on);

– Statins or other lipid-lowering agents;

– Beta-blockers;

– Angiotensin converting enzyme inhibitors;

– Angiotensin-II receptor antagonists;

– Insulin and oral hypoglycaemic agents;

– Medications for the treatment of IC (Pentoxifylline, Buflomedil).

• Baseline anatomic characteristics of the target lesion in the common iliac artery, as determined by DSA and pre-operative CT-angiography or MR-angiography:

– Arterial inflow: Impaired inflow is defined as presence of hemodynamically significant obstruction in the aorta;

– Arterial outflow: Impaired outflow is defined as presence of hemodynamically significant obstruction in the external iliac artery, common femoral artery or both superficial and deep femoral arteries. Isolated hemodynamically significant obstruction in either the superficial or deep femoral artery will not be considered impaired outflow;

Reference vessel diameter, obtained from averaging 5mm segments proximal and distal to the lesion;

Involvement of origin/ostium;

Lesion length, documented in centimeters and classified as follows:

▪ Focal: ≤1 cm;

▪ Short: >1 and <3 cm;

▪ Intermediate: ≥ 3 and < 5 cm;

▪ Long: ≥ 5 cm;

▪ In case of vessel occlusion with a stenosed segment, both the length of the stenosed segment and the length of the occluded segment should be reported.

– Occlusion or stenosis

– In case of stenosis

▪ Minimal luminal diameter;

▪ Percent diameter stenosis;

▪ Systolic pressure gradient, measured as described previously.

– Calcification: Semi-quantitative distinction between no, moderate, and heavy calcification at the site of the lesion.

– TASC-classification.

• Baseline anatomic characteristics of other hemodynamically significant lesions at the aortoiliac and femoropopliteal level:

– Arterial segment(s):

▪ Infra-renal abdominal aorta;

▪ Internal iliac artery;

▪ External iliac artery;

▪ Common femoral artery;

▪ Deep femoral artery;

▪ Superficial femoral artery;

▪ Popliteal artery.

– Lesion length, documented in centimeters and classified as follows:

▪ Focal: ≤1 cm;

▪ Short: >1 and <5 cm;

▪ Intermediate: ≥ 5 and < 15 cm;

▪ Long: ≥ 15 cm;

▪ In case of vessel occlusion with a stenosed segment, both the length of the stenosed segment and the length of the occluded segment should be reported.

– Occlusion or stenosis

• Crural outflow: Each of the crural arteries with patency directly to the foot will score 1 point. A patent dorsal and plantar pedal arch will each score 1 point. This will lead to a score of 0 to 5, indicating crural outflow. As it is not standard procedure to make a DSA of the crural arteries during iliac interventions, pre-operative CT-A or MR-A will be used to score this.

• Presence of multilevel disease: Defined as presence of significant obstructive lesions at more than one level in the same limb (aortoiliac, femoropopliteal and crural). When patients are included with both limbs, one limb can have multilevel disease, while the other limb does not.

• Disease-related health status and quality of life: As defined by the Walking Impairment Questionnaire (WIQ) and RAND-36 questionnaire.

• Rutherford stage.

• Functional status:

– In patients with IC: Claudication onset time (COT) and absolute claudication time (ACT) on a standardized treadmill test (3.2 km/h at a 12% grade for a maximum of 5 minutes).

• Hemodynamic status:

– In patients with IC: ABI at rest and after standardized treadmill test;

– In patients with CLI: Systolic ankle pressure and ABI.

• Degree of stenosis and peak systolic velocity ratio on DUS.

#### Procedure-related parameters

• Grams of intravenous iodine used for contrast. The amount of millilitres used will be scored during the procedure, and post-hoc the amount of iodine will be calculated. In our centre, we use Visipaque®, which contains 320mg of Iodine per ml

• Radiation time and dosage, as is measured by the fluoroscopy device

• Amount and type of materials used (guidewires, sheaths, balloons, catheters)

• Type, diameter, number and length of stents used

• Lesion characteristics after treatment:

– Arterial inflow: Impaired inflow is defined as presence of hemodynamically significant obstruction in the aorta;

– Arterial outflow: Impaired outflow is defined as presence of hemodynamically significant obstruction in the external iliac artery, common femoral artery or both superficial and deep femoral arteries. Isolated hemodynamically significant obstruction in either the superficial or deep femoral artery will not be considered impaired outflow;

– Minimal luminal diameter (MLD);

– Percent diameter stenosis;

– Systolic pressure gradient.

• Peri-procedural complications.

• Technical success

• Device success

#### Parameters scored during regular follow-up

• Disease-related health status and quality of life: As defined by the Walking Impairment Questionnaire (WIQ) and RAND-36 questionnaire.

• Rutherford stage.

• Functional status:

– In patients with IC: Claudication onset time (COT) and absolute claudication time (ACT) on a standardized treadmill test (3.2 km/h at a 12% grade for a maximum of 5 minutes).

• Hemodynamic status:

– In patients with IC: ABI at rest and after standardized treadmill test;

– In patients with CLI: Systolic ankle pressure and ABI.

• Degree of stenosis and peak systolic velocity ratio on DUS.

• Occurrence of amputation.

• Mortality.

#### Parameters scored in case of restenosis or reocclusion

• Anatomic characteristics of the restenosis or occlusion in the stent in the common iliac artery:

– Arterial inflow: Impaired inflow is defined as presence of hemodynamically significant obstruction in the aorta.

– Arterial outflow: Impaired outflow is defined as presence of hemodynamically significant obstruction in the external iliac artery, common femoral artery or both superficial and deep femoral arteries. Isolated hemodynamically significant obstruction in either the superficial or deep femoral artery will not be considered impaired outflow.

– Relation of stenosis or occlusion to the stent:

▪ Proximal edge, defined as 1 cm proximal and distal to the proximal edge of the stent;

▪ In-stent, defined as 1 cm distal from the proximal to 1 cm proximal from the distal end;

▪ Distal edge, defined as 1 cm proximal and distal to the distal edge of the stent.

– Lesion length, documented in centimeters and classified as follows:

▪ Focal: ≤1 cm;

▪ Short: >1 and <3 cm;

▪ Intermediate: ≥ 3 and < 5 cm;

▪ Long: ≥ 5 cm;

▪ In case of vessel occlusion with a stenosed segment, both the length of the stenosed segment and the length of the occluded segment should be reported.

– Occlusion or stenosis;

– In case of stenosis:

▪ Minimal luminal diameter;

▪ Percent diameter stenosis;

▪ Systolic pressure gradient.

• Procedural success rate of re-intervention.

### Randomization, blinding and treatment allocation

**Figure 2:**
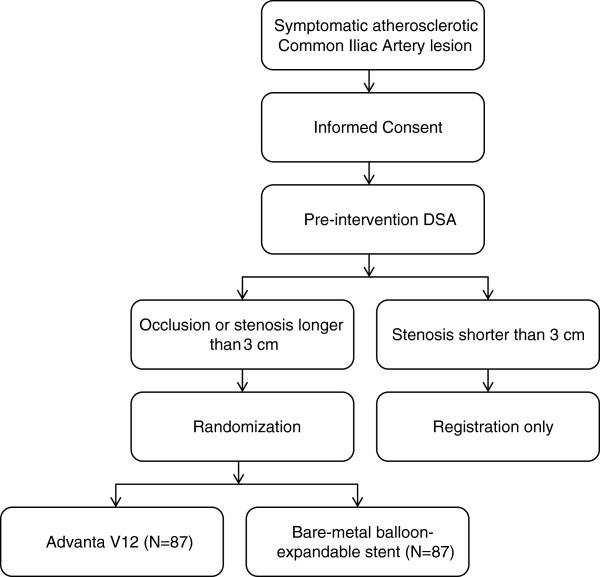
Flowchart describing the inclusion and randomization of patients.

All symptomatic patients in whom a hemodynamically significant lesion of the CIA is suspected will be included. This is assessed using DUS and either CT-angiography or MR-angiography. A hemodynamically significant lesion is suspected if DUS shows a peak systolic velocity ratio of ≥2.4 and/or CT-angiography or MR-angiography shows a diameter reduction of ≥50%. Any suspected significant lesion is initially included, and informed consent obtained, even if pre-operative imaging shows a stenosis with a length of less than 3 cm. After the pre-intervention DSA and intra-arterial systolic blood pressure gradient measurement have been performed, the treating physician will make the final decision if the patient meets the inclusion criteria. If there is a stenosis with a length of more than 3 cm, or an occlusion, the patient will be randomized. All patients with stenoses with a length of less than 3 cm will be excluded, but they will be registered, and informed consent will have been obtained to store their data (Figure 2). Randomization will be performed using an online registration and randomization program (Trans European Network for Clinical Trial Services (TENALEA), http://tenalea.net). Unstratified randomization will be used, with a minimization algorithm based on occlusion vs stenosis, and endovascular vs hybrid procedures. If a patient has bilateral iliac lesions that are eligible, the symptomatic limb will be included. In case both limbs are symptomatic, the leg with the lowest ABI will be included. Both legs will receive the same treatment. The patient will be allocated to either the Advanta V12 PTFE-covered stent, or to an uncovered balloon-expandable stent. Patients will be blinded for the treatment they receive. When patients are under local or regional anesthesia, a sterile operating theatre napkin will be placed in such a manner that the patient cannot see the procedure and the stent in particular. The physician will make sure not to mention the type of stent prior, during or after the procedure. The physician performing the procedure will not be blinded, as this is not possible practically. The type of stent used will not be documented in the patient’s medical record. Post-operative ABI’s, DUS and scoring of Rutherford classification will be performed by vascular technicians who are blinded for the type of stent used. Therefore, this is a double-blind study.

In both centers, we have, as a standard procedure, a separate file in which the product sticker of every placed stent is put, coupled with the name, birth date and patient number of the patient that received the stent. Neither the patient, nor the personnel who perform post-operative investigations, nor the analyst analyzing the data have insight in to this file; therefore keeping this file does not threaten the double-blind nature of this study.

The indication for breaking the randomization code is the need for open surgical or endovascular re-intervention if the treating physician feels this is required. The physician will then contact the coordinating or one of the principal investigators, who will reveal the type of stent and document breaking of the randomization code. Alternatively, this can be looked up in the separate file described in the previous paragraph.

### Study procedures

#### Digital Subtraction Angiography (DSA)

This is a widely used diagnostic technique to visualize peripheral arteries using fluoroscopy. A pre-contrast image is made, after which intra-arterial contrast is given proximal to the area that needs to be visualized. Using digital techniques, the pre-contrast image is subtracted from later images, thereby visualizing the degree of contrast in the arterial lumen without interference from bony or soft tissue structures.

#### Duplex Ultrasonography

This is a non-invasive diagnostic technique to estimate stenoses or occlusions in peripheral arteries. It incorporates two elements:

• Grayscale: Uses ultrasound to directly visualize the structure of the vessel. No motion or bloodflow is assessed. In this way, stenosis or occlusion can be directly visualized

• Color-doppler: Uses ultrasound to visualize the flow in the vessel. This is expressed as peak-systolic velocity (PSV), the highest velocity of the blood, during the systolic phase, in cm/sec. In a stenosis, the PSV will increase. When a stenosis is suspected, the peak-systolic velocity ratio is calculated. This is defined as the ratio of intra-stenotic PSV to pre-stenotic PSV. A ratio of >2.4 will be defined as a significant stenosis. When the vessel is occluded, no flow will be measured.

All study-related DUS are required to be performed in the vascular lab of the hospital by dedicated laboratory personnel who are blinded for the type of stent used.

#### ABI measurement

Systolic pressure in both arms is measured, using an automatic blood pressure monitor. The higher of the two will be used. Next, a blood pressure cuff is placed around the patient’s calf. Using a Doppler ultrasound device the dorsal pedal artery and posterior tibial artery are located. The blood pressure cuff is inflated until the Doppler signal disappears, and then slowly deflated. The pressure at which a Doppler signal reappears is documented for both arteries. The higher of the two is then divided by the systolic pressure in the arm. This is the ABI. When a standardized treadmill test (in patients with IC) is performed, this ABI measurement will be performed prior to the test (ABI in rest) and after completion of the test (ABI after exercise). All study-related ABI measurements are required to be performed in the vascular lab of the hospital by dedicated laboratory personnel who are blinded for the type of stent used.

#### Standardized treadmill test

Patients are required to walk at 3.2 km/h at a 12% grade for a maximum of 5 minutes on a treadmill. Claudication onset time (COT) will be measured (in minutes and seconds), which is defined as the time after initiation of exercise when the patients first experiences symptoms of claudication, and absolute claudication time (ACT), which is defined as the time after initiation of exercise until the patient cannot continue due to severe claudication pain. Patients with CLI are not required to undergo the standardized treadmill test. All study-related treadmill tests are required to be performed in the vascular lab of the hospital by dedicated laboratory personnel who are blinded for the type of stent used.

#### Walking Impairment Questionnaire (WIQ)

The WIQ is a short, easy to fill out 14-item questionnaire that evaluates the degree of walking impairment in patients with intermittent claudication on three domains; walking distance, walking speed and the ability to climb stairs. These three domains represent common daily activities that are frequently impaired in patients with symptomatic PAOD and contribute to the degree of walking impairment. A Dutch translation, using the metric system, has been validated [57].

#### RAND-36 questionnaire

The RAND-36 questionnaire is a short, easy to fill out 36-item questionnaire, designed for scoring general quality of life and health status [58]. It is identical to the SF-36, but uses a slightly different scoring algorithm [59]. A translated Dutch version has been made, which has been validated [60]. It covers eight domains, and assesses these domains over the past 4 weeks. Domains can score from 0 to 100 points, with higher scores representing less physical limitation, fewer symptoms, greater treatment satisfaction, or better quality of life. The domains that are tested are: physical functioning, social functioning, physical role impairment, emotional role impairment, vitality, pain, mental health and general health perception. A separate question assesses health change with standardized response choices.

### Follow up scheme

During follow-up, any change in baseline characteristics, complication, or newly developed (re)stenosis or (re)occlusion will have to be documented as described in ’Endpoints/parameters’.

#### Pre-operative

Pre-operative, DUS will be performed to document the occlusion or degree of the stenosis in the iliac and common femoral artery. This is mandatory, as this is the modality that will be used during follow-up. Additionally, either CT-angiography (CT-A) or MR-angiography (MR-A) of the entire lower extremity must be performed at the physician’s discretion. An ABI will be measured, including a treadmill test in claudicants. These are all part of our regular pre-operative work-up. In most cases, one or more of these investigations will have been performed prior to inclusion. When this has been no longer than 3 months prior to inclusion, the investigation will not have to be repeated for study purposes. All other relevant study parameters will be documented and patients will be asked to fill in the RAND-36 and WIQ.

#### Per-operative

Prior to placement of the stent, a DSA in two imaging planes, with at least 30, but preferably more than 45 degrees difference in rotation will be performed, and a systolic pressure gradient will be measured. After successful stent placement, a DSA (in two imaging planes) and systolic pressure gradient will once again be performed. If open surgical and/or endovascular repair of other arteries of the lower extremity was performed, this will be documented. Any complication that occurred during the procedure will be documented.

#### 1 month postoperative

At 1 month, patients will undergo an ABI with treadmill test (patients with persistent CLI will only require ABI measurement in rest). Any complication that occurred within the first 30 days will be scored. Patients will be asked to fill in the RAND-36 and the WIQ.

#### 6, 12 and 24 months postoperative

At 6, 12 and 24 months, ABI and treadmill test will be performed, as well as a DUS, and patients will be asked to fill in the RAND-36 and WIQ. If DUS shows a stenosis, defined as a peak systolic velocity rate of ≥2.4, or is inconclusive, CT-A, MR-A or DSA will be performed. If an in-stent stenosis or occlusion is identified, this will be documented and treated within 1 month. Table 3 summarizes the study procedures at different moments.

**Table 3:** Study procedures

	**Pre-ope-rative**	**Per-ope-rative**	**1 month**	**6 months**	**12 months**	**24 months**	**In case of restenosis**
DUS	X			X	X	X	X
ABI (with or with-out treadmill test)	X		X	X	X	X	X
DSA		X					X
WIQ/RAND-36	X		X	X	X	X	

DUS = Duplex ultrasonography, ABI = Ankle-brachial index, DSA = Digital subtraction angiography, WIQ = Walking Impairment Questionnaire, RAND-36 = RAND 36-Item Health Survey.

#### Unplanned visits

Patients might present themselves at the outpatient clinic or the emergency ward in between study visits, with (recurrent) symptoms and complaints of PAOD. In this case at least ABI in rest and DUS is mandatory. If DUS shows a stenosis, defined as a peak systolic velocity rate of ≥2.4, or is inconclusive, CT-A, MR-A or DSA will be performed. If an in-stent stenosis or occlusion is identified, this will be documented and treated within 1 month. Patients will then resume their regular follow-up scheme as planned.

### Withdrawal of individual subjects

Subjects can leave the study at any time for any reason if they wish to do so without any consequences. The investigator can decide to withdraw a subject from the study for urgent medical reasons. This will be recorded in the CRF and the physician is required to notify the Principal Investigator.

### Replacement of individual subjects after withdrawal

Patients that have been randomized and are withdrawn from the study will be not replaced. They will be considered lost to follow-up and included in the data analysis based on intention-to-treat. Patients that have been included but are withdrawn prior to randomization will be replaced. This will be documented.

### Follow-up of subjects withdrawn from treatment

Patients that for whatever reason receive a different treatment than randomized, or received no treatment will be followed-up and included in the trial, based on intention-to-treat. Patients that are withdrawn from follow-up will be considered lost to follow-up.

### Premature termination of the study

If at any point during the study information becomes available, either from interim analysis of results, or from external sources, that the investigated device poses an increased risk for participating patients, the study will be terminated. Patients will be unblinded for the type of stent they received. If applicable, they will receive additional treatment or intervention.

### Safety reporting

#### Section 10 WMO event

In accordance to section 10, subsection 1, of the WMO, the investigator will inform the subjects and the reviewing accredited medical ethical review committee (METC) if anything occurs on the basis of which it appears that the disadvantages of participation may be significantly greater than was foreseen in the research proposal. The study will be suspended pending further review by the accredited METC, except insofar as suspension would jeopardize the subjects’ health. The investigator will take care that all subjects are kept informed.

#### Adverse and serious adverse events

Adverse events are defined as any undesirable experience occurring to a subject during the clinical trial, whether or not considered related to the investigational treatment. All adverse events reported spontaneously by the subject or observed by the investigator or his staff will be recorded.

A serious adverse event is any untoward medical occurrence or effect that:

• Causes death, or is life threatening (at the time of the event);

• Requires hospitalization or prolongation of existing inpatients’ hospitalization;

• Results in persistent or significant disability or incapacity.

All SAEs will be reported by the responsible investigator through the web portal ToetsingOnline to the accredited METC that approved the protocol within 15 days after the sponsor has first knowledge of the serious adverse reactions.

SAEs that result in death or are life threatening should be reported expeditiously. The expedited reporting will occur not later than 7 days after the responsible investigator has first knowledge of the adverse reaction. This is for a preliminary report, with another 8 days for completion of the report.

#### Follow-up of adverse events

All adverse events will be followed-up until they have abated, or until a stable situation has been reached. Depending on the event, follow up may require additional tests or medical procedures as indicated, and/or referral to a general physician or a medical specialist

#### Data safety monitoring board

Following the recommendations of the ’Quality assurance in human medical research’ charter (Dutch: Kwaliteitsboring van mensgebonden onderzoek) by the ‘Dutch Federation of Academic Medical Centers’ (Dutch: Nederlandse Federatie van Universitair Medische Centra), we do not consider the formation of a Data Safety Monitoring Board (DSMB) sensible. A DSMB has several functions. First, is to assess whether statistical significance can still likely be reached or, on the other hand, may be reached preliminarily for the main outcomes, and thus whether the study group size should be changed. However, our main outcomes are all scored at 2 years. So, when a relevant group has reached the 2-year follow-up, and an interim analysis would be sensible, we will already have included all of our patients. So, the interim analysis would not contribute anything on that field.

A second function of the DSMB is to assess the safety and (S)AE’s of the investigational products. However, all stents that are used in the trial are registered for use in the iliac artery and have been used for years. As well, virtually all complications are procedure-related and not stent-related. Therefore, a difference in safety or occurrence of (S)AE’s is unlikely. Of course, the investigators and the METC will monitor the occurrence and rate of (S)AE’s.

### Statistical analysis

Statistical analysis will be performed for the complete study population, as well as subgroup analysis for patients with IC vs CLI, occlusions vs stenoses, and endovascular vs hybrid procedures.

#### Descriptives

Descriptive analysis will be performed to compare the baseline characteristics of both groups.

• Continuous data (age, BMI, length, and so on): Averages with standard deviation (parametric data) or medians with percentiles (non-parametric data) will be calculated for both groups.

• Categorical data (TASC type, co-morbidities, and so on): Frequencies will be calculated for both groups.

Distribution of Rutherford categories during baseline and follow-up will be given as percentages per category. No statistical analysis will be performed.

#### Comparative statistics

The primary and secondary endpoints as mentioned in ’Endpoints/parameters’ will be compared using statistical methods.

#### Actuarial analysis

Absence of binary restenosis rate and reocclusion rate will be calculated with actuarial (life-table) analysis and will be expressed as a percentage with a standard error and 95% CIs. Logrank tests will be used to determine if differences between the estimates are significant. For these endpoints, we will also calculate a combined endpoint with survival, for example, binary restenosis-free survival etc. Based on the literature, we expect that mortality will be negligible and will not influence the results, and these separate analyses will be performed to check this assumption.

#### Univariate analysis

Univariate analysis will be performed to assess differences in the other aforementioned primary and secondary endpoints.

Continuous data: Averages with standard deviation (parametric data) or medians with percentiles (non-parametric data) will be calculated. Differences between both groups will be tested using a Student’s *t*-test (parametric data) or a Mann–Whitney *U*-test (non-parametric data). Differences with a *P*-value <0.05 will be considered statistically significant. The following endpoints will be assessed:

Post-intervention COT and ACT;

Mean ABI.

Categorical data: Frequencies will be calculated and difference between both groups will be tested using Chi-squared test. Differences with a *P*-value <0.05 will be considered statistically significant. The following secondary endpoints will be assessed:

• Target lesion revascularization rate and target extremity revascularization rate;

• Procedural, technical and device success rate;

Mortality rate, complication rate, amputation rate;

• Rate of device-specific problems.

#### Interim analysis

Interim analysis is not deemed sensible, as described earlier.

#### Software

All statistical analysis will be performed with the Statistical Package for the Social Sciences (SPSS) version 16.0 or up (IBM, Armonk, NY, USA).

### Ethical considerations

#### Regulation statement

This study will be conducted according to the principles of the Declaration of Helsinki (version: October 2008) and in accordance with the Medical Research Involving Human Subjects Act (Dutch: Wet Medisch-wetenschappelijk Onderzoek met mensen; WMO).

#### Recruitment and consent

All patients who present at the outpatient clinic or emergency ward with symptomatic PAOD of the common iliac artery will be considered possibly eligible for inclusion. The present surgical or radiological resident or staff member will screen the patient for meeting the in- and exclusion criteria. If for whatever reason the physician at this moment does not screen the patient, any other involved physician or one of the investigators may include the patient at a later moment. When the patient meets the inclusion criteria, the physician will then inform the patient of the study, including the risks and burdens as mentioned before and the availability of an independent physician. A written information letter will be presented to the patient to read, this will include contact details of an independent physician. The patient will then be offered the opportunity to ask questions. Patients will be offered 24 hours to consider their decision. When they agree to participate in the study, they will be asked to fill out an informed consent form. Both the information letter and the informed consent form are attached as separate documents to this protocol.

#### Benefits and risks assessment

Participating patients will need to make five study-related hospital visits. One CT-angiography or MR-angiography, four DUS and five ABI measurements with treadmill tests (only in patients with IC) will be performed. When compared to our standard work-up and follow-up, patients need to make two extra hospital visits, and will receive two extra DUS and three extra ABI measurements with treadmill test. When DUS shows possible restenosis, patients will receive additional DSA, CT-angiography or MR-angiography. This is standard procedure for all patients. Patients will also be asked to fill in two questionnaires five times. Patients who participate may benefit by being treated with a stent that may have a lower restenosis rate.

#### Compensation for injury

The investigator has an insurance which is in accordance with the legal requirements in the Netherlands (Article 7 WMO and the Measure regarding Compulsory Insurance for Clinical Research in Humans of 23 June 2003). This insurance provides cover for damage to research subjects through injury or death caused by the study.

This insurance was taken out with:

Medirisk

Atoomweg 100

3542 AB Utrecht

The Netherlands (+31) 030–241133

The maximum coverage of this insurance is:

1. € 450.000,- (that is, four hundred and fifty thousand Euro) for death or injury for each subject who participates in the Research;

2. € 3.500.000,- (that is, three million five hundred thousand Euro) for death or injury for all subjects who participate in the Research;

3. € 5.000.000,- (that is, five million Euro) for the total damage incurred by the organization for all damage disclosed by scientific research for the Sponsor as ‘verrichter’ in the meaning of said Act in each year of insurance coverage.

The insurance applies to the damage that becomes apparent during the study or within 4 years after the end of the study.

#### Incentives

Participating patients will not receive any incentives or compensation.

### Administrative aspects and publication

#### Handling and storage of data and documents

Data will be documented using an online interface and will be stored on a separate server. This database and server was created for the DEALL (Dutch Endovascular ALLiance) research group, and use of this database and server was previously approved by the board of the Maasstad Hospital Rotterdam and Sint Antonius Hospital Nieuwegein for collection and storage of observational data. Only the coordinating and principal investigators per site will have access to the source data.

All data will be handled anonymously. Each participant will receive a study number, consisting of the letters DIS followed by three numbers. These numbers will be awarded increasingly, according to time of inclusion. So, the first participant will receive DIS-001, and so on. This number will be used in data analysis or any publications or reports on the trial. Only the coordinating investigator and principal investigators will have access to the key file which contains the coupling of the study number with patients personal data.

The handling of personal data complies with the Dutch Personal Data Protection Act (in Dutch: De Wet Bescherming Persoonsgegevens, Wbp).

#### Amendments

Amendments are changes made to the research after a favorable opinion by the accredited METC has been given. All amendments will be notified to the METC that gave a favorable opinion.

#### Annual progress report

The sponsor/investigator will submit a summary of the progress of the trial to the accredited METC once a year. Information will be provided on the date of inclusion of the first subject, numbers of subjects included and numbers of subjects that have completed the trial, serious adverse events/serious adverse reactions, other problems, and amendments.

#### End of study report

The investigator will notify the accredited METC of the end of the study within a period of 8 weeks. The end of the study is defined as the last patient’s last visit.

In case the study is ended prematurely, the investigator will notify the accredited METC, including the reasons for the premature termination.

Within one year after the end of the study, the investigator/sponsor will submit a final study report with the results of the study, including any publications/abstracts of the study, to the accredited METC.

#### Public disclosure and publication policy

After acceptance of the study protocol, the study protocol will be submitted to a peer-reviewed journal that publishes study protocols. The first journal that it will be submitted to is the open access, peer-reviewed, online journal *Trials*. The trial will also be registered in the Dutch Trial Register (NTR, in Dutch: Nederlands Trial Register),

After completion of the trial, results, regardless of the outcome, will be submitted for publication in a peer-reviewed journal.

## Trial status

Inclusion of patients will commence 1 May 2012.

## Definitions

ABI, Ankle-brachial index. Defined as the ratio between the highest systolic pressure in both brachial arteries and the highest systolic pressure in the crural arteries. In patients without CLI this is repeated after the standardized treadmill test, termed ‘ABI in rest’ and ‘ABI after exercise’; ABR form, General Assessment and Registration form, is the application required for submission to the accredited Ethics Committee (in Dutch: Algemene Beoordeling en Registratie); Absence of binary restenosis, Absence of hemodynamically significant obstruction (see definition) after endovascular intervention; ACT, Absolute claudication time. Defined as the time (in minutes and seconds) after initiation of the treadmill test when the patient cannot walk any further due to severe the patient cannot walk any further due to severe claudication pain; Aortoiliac level, Proximal limit: Origin of the renal arteries. Distal limit: Deep circumflex iliac artery inguinal ligament; COT, Claudication onset time. Defined as the time (in minutes and seconds) after initiation of the treadmill test when patient first experiences symptoms of claudication; Crural level, Proximal limit: Origin of anterior tibial artery.Including foot arteries; DSA, Digital subtraction angiography. Before and after every intervention a DSA of the aortoiliac level will be performed and saved. DSA will be performed in two imaging planes with at least 30 but preferably more than 45 degrees difference in rotation. DSA will be performed according to a standard protocol; Femoro-popliteal level, Proximal limit: Deep circumflex iliac artery inguinal ligament. Distal limit: Origin of anterior tibial artery; Hemodynamically significant obstruction, Hemodynamic significance can be assessed using the following measurement techniques (in hierarchical order):Intra-arterial translesional systolic blood pressure gradient >10 mmHg as assessed by using a 70cm straight catheter (4F) without side holes which is withdrawn through the stenosis. Intravascular ultrasound (IVUS) measurements indicating ≥50% diameter stenosis. Intra-arterial DSA,indicating ≥50% diameter stenosis. CT-angiography or MR-angiography indicating ≥50% diameter stenosis. DUS indicating ≥50% diameter stenosis as defined by a peak systolic velocity ratio >2.4 (Defined as the ratio of intra-stenotic peak systolic velocity to pre-stenotic peak systolic velocity); MLD, Minimal luminal diameter. The minimal luminal diameter in a stenosis as measured by DSA; Standardized treadmill test, Patients are required to walk with 3.2 km/h at a 12% grade for a maximum of 5 minutes on a treadmill. Claudication onset time (COT) and absolute claudication time (ACT) are determined in minutes and seconds.

## Abbreviations

AE: Adverse Event; AIOD: Aorto-iliac occlusive disease; CCMO: Central Committee on Research Involving Human Subjects; in Dutch: Centrale Commissie Mensgebonden Onderzoek; CIA: Common Iliac Artery; CLI: Critical limb ischemia (Rutherford category 4 5 or 6); DUS: Duplex ultrasonography; EU: European Union; GCP: Good Clinical Practice; IC: Informed Consent; METC: Medical research ethics committee (MREC) in Dutch: medisch ethische toetsing commissie; PAOD: Peripheral arterial occlusive disease; PTFE: Polytetrafluoroethylene; (S)AE: (Serious) Adverse Event; TER: Target-extremity revascularization; TLR: Target-lesion revascularization; WMO: Medical Research Involving Human Subjects Act (in Dutch: Wet Medisch-wetenschappelijk Onderzoek met Mensen; Wbp: Personal Data Protection Act (in Dutch: Wet Bescherming Persoonsgevens).

## Competing interests

An unrestricted grant for this study was received from Atrium Medical Inc. (Hudson, NH, USA). The author(s) declare that they have no competing interests.

## Authors’ contributions

JB performed a literature search for the protocol’s background paragraph. All authors contributed to establishing the objectives, methods and design of the trial. All authors have made significant suggestions and contributions to the protocol. All authors have given approval to the final protocol. BF is coordinating investigator/project leader. JAV and JB are principal investigators of their trial sites. All authors read and approved the final manuscript.

## Authors’ information

JB is a surgical resident at Maasstad Ziekenhuis, Rotterdam, The Netherlands. BF and JPV are vascular surgeons at Maasstad Ziekenhuis, Rotterdam, The Netherlands and St. Antonius Ziekenhuis, Nieuwegein, The Netherlands, respectively. RA and JAV are interventional radiologists at Maasstad Ziekenhuis, Rotterdam, The Netherlands and St. Antonius Ziekenhuis, Nieuwegein, The Netherlands, respectively. All authors are members of the Dutch Endovascular Alliance (DEALL). This is a collaboration of vascular surgeons and interventional radiologists, aiming to perform scientific research regarding endovascular treatment of vascular disease.
